# Advancing paediatric nutrition: interdisciplinary perspectives from the clinic to the community – symposium proceedings

**DOI:** 10.1186/s12919-026-00395-z

**Published:** 2026-10-06

**Authors:** Shalini G. Hegde, Greeshma Mulakatte, Alekia Uscoicar, Anura V. Kurpad, Pratibha Dwarkanath

**Affiliations:** 1 Department of Paediatric Surgery, St. John’s Medical College Hospital, St. John’s National Academy of Health Sciences, Bengaluru, 560034 India; 2 Division of Nutrition, St. John’s Research Institute, St. John’s National Academy of Health Sciences, Bengaluru, 560034 India; 3 Department of Physiology, St. John’s Medical College, St. John’s National Academy of Health Sciences, Bengaluru, 560034 India

**Keywords:** Paediatric nutrition, Short bowel syndrome, Interdisciplinary, Low-and-middle-income countries

## Abstract

Paediatric nutrition directly influences survival, clinical outcomes, and long-term health, and is particularly of significance in India with one of the world’s largest child populations. This symposium, hosted by St. John’s National Academy of Health Sciences as part of an ICMR-funded Centre for Advanced Research initiative, convened national and international experts spanning from clinical nutrition and public health. Day 1 focused on the management of intestinal failure and the life-saving role of parenteral and enteral nutrition, highlighting global best practices, surgical advances, and the need to strengthen multidisciplinary nutrition support teams in India. Day 2 shifted to maternal and child nutrition at the community level, addressing breastfeeding challenges, complementary feeding, stunting, and government program strategies. Together, the sessions underscored that nutrition for vulnerable children requires continuous care supported by technology, coordinated systems, and policy. The symposium laid the foundation for sustained annual engagement to strengthen paediatric nutrition systems in India.

## Introduction

Paediatric nutrition lies at the heart of improved clinical outcomes and public health progress, directly influencing a child’s survival, growth, neurodevelopment, and lifelong health trajectory. With children aged 0–19 y projected to represent 32% of India’s total population in 2026, the nation is home to one of the largest paediatric populations in the world [1]. This vast demographic underscores not only the responsibility but also the promising opportunity to strengthen nutrition care and ensure a healthier future for every child. While the country has made notable strides in maternal and child health, undernutrition, micronutrient deficiencies, rising childhood obesity [2] and the double burden of malnutrition [3, 4] continue to challenge health systems. These threats are magnified in children with complex medical and surgical conditions, for whom nutrition is not merely supportive care but a true lifesaving intervention.

For clinical conditions, the ability to provide long term parenteral nutrition (PN) [5, 6] gave a new lease of life to children who were premature with low birth weight, necrotizing enterocolitis and paediatric surgical conditions like intestinal atresia and total colonic aganglionosis [7, 8]. Despite widespread use of PN globally since the 1960 s, India continues to underutilize this therapy. Practical barriers including limited availability of standardized formulations, apprehension regarding infection or metabolic complications, cost considerations, and lack of training continue to restrict access. Simplifying PN delivery systems, strengthening multidisciplinary teamwork, and promoting clinician education are essential steps in overcoming these longstanding challenges.

The primary objective of this symposium was to unite professionals across the spectrum of paediatric nutrition on a single platform to foster meaningful discussion, collaboration and teamwork. After all, it takes a village to feed a child. The symposium aimed to showcase cutting edge advances in both the clinical care and public heath aspects of paediatric nutrition, delivered by leading national and international experts. The first day focused on the comprehensive management of short bowel syndrome a complex clinical condition that demands the coordinated expertise of paediatric surgeons, gastroenterologists, intensivists, dietitians, clinical pharmacists, and specialized nursing teams. The second day shifted the spotlight to public health and community-based child nutrition, emphasizing strategies to improve nutritional status on a population scale through the combined efforts of diverse stakeholders and disciplines.

## Methods

### Institutional framework

The Division of Nutrition, in collaboration with the, Department of Paediatric Surgery of St John’s National Academy of Health Sciences (SJNAHS), Bengaluru, India, hosted the symposium titled *“Advancing Paediatric Nutrition: Interdisciplinary Perspectives from the Clinic to the Community”.* This first of its kind symposium was designed for knowledge dissemination across all disciplines and will evolve as an annual event, expanding next year to include focused training workshops. This event was held as a flagship initiative of the Centre for Advanced Research grant awarded by the Indian Council of Medical Research (ICMR- CAR) to the institution (2025–2030) for research in maternal and child health. The investigators of this grant are Dr Pratibha Dwarkanath (Principal Investigator), Dr Shalini Hegde, Dr Tinku Thomas, Dr Ambily Sivadas and Dr Anura Kurpad from SJNAHS with collaborators Dr Ranadip Chowdhury and Dr Sunita Taneja from the Society of Applied Sciences, New Delhi. This large-scale academic event was made possible by the valuable support by the Directorate of SJNAHS who provided their well-established facilities like the venue hall, catering and on campus accommodation for the symposium and were an integral part of the inaugural function held on Day 1.

### Organizational framework

To ensure participation from multiple disciplines, informational flyers featuring the symposium theme and agenda were circulated via social media starting from September 1 st, 2025. This outreach was carried out in collaboration with the Karnataka State Chapter of the Indian Association of Paediatric Surgeons, Indian Association of Parenteral and Enteral Nutrition (IAPEN) India, Indian Dietetic Association (IDA) Bengaluru chapter to maximize engagement and attendance.

### Scientific programme development

A carefully curated list of potential speakers was prepared, prioritizing individuals with recognized expertise across relevant clinical, surgical and nutritional disciplines. The invitation list included leading national faculty and well as distinguished international experts to bring global perspectives to the discussions. International experts were invited from Boston Children’s Hospital who have set benchmarks in the management of short bowel syndrome and intestinal failure through a collaborative, multidisciplinary model of care. Invitations were also extended to the team at Sidra Hospital, Qatar which runs an intestinal rehabilitation program in the Middle-East. The participating pediatric surgeon an alumnus of SJNAHS brought valuable insights grounded in an understanding of the prevailing health care system, supporting ongoing efforts to develop short bowel services in India. Invited speakers who confirmed their participation were subsequently provided with thematic objectives along with a structured presentation format guide to ensure alignment with the symposium goals.

### Symposium delivery

The symposium was conducted in hybrid mode from 9:00 to 17:30 IST on October 17th and 18th 2025. The in-person sessions were conducted in Lecture hall—1, Golden Jubilee Block, St. John’s Medical College Building, Koramangala, Bengaluru, India while the online session was hosted via ‘Zoom Webinar’. For the registration, a nominal fee was charged. The offline registration portal was opened from September 9th to October 15th, 2025 followed by a short window for online registrations which remained opened for 24 h from 16th October, 2025 to the start of the event.

Approximately 220 participants registered for the in-person event, with few additional participants attending online from 3 countries—India, Qatar and USA. Forty percent of the registrations were doctors, 30% included dietitians and public health nutritionists, 15% each were nurses and clinical pharmacists.

The symposium agenda (Table 1) encompassed several segments, an inaugural function, expert speaker presentations, panel discussions, concluding remarks and was awarded four Continuing Medical Education credit points from the Karnataka Medical Council for those holding a medical license.

**Table 1:** Symposium Agenda

**Day 1–17th October 2025**					
Time	Session	Speaker(s)			Chairperson/Moderator (s)
8:50–9:00 AM	Welcome Note	Dr. Prasanna Kumar			
9:00–9:30 AM	The National Scene on Paediatric Nutrition	Dr. Sarath Gopalan			Dr. Harshad Devarbhavi Dr. Gowri Shankar
9:30–10:00 AM	Paediatric Clinical Nutrition & Short Bowel Syndrome	Dr. Dhanasekar Kesavelu			Dr. Sanjay Rao Dr. Shilpa Varma
10:00–10:30 AM	Inauguration & Group Photo				
10:30–10:45 AM	Tea Break				
10:45–11:45 AM	Lines, Tubes, STEPS – The Surgeon’s Role in Supporting Nutrition		Dr. Biren Modi		Dr. Ramesh S Dr. Padmalatha K
11:45–1:30 PM	Panel Discussion – Long term Parenteral Nutrition		Dr. Biren Modi Dr. Anand Alladi Dr. Amit Yelsangikar Dr. Shubha AM		Moderator- Dr. Saleem Mammoo
	Parenteral Nutrition Compounding		Dr. Gauri Krishna Dr. Sanjay Rao Dr. Antony Robert Dr. Manjiri S		Moderator- Dr. Saleem Mammoo
1:30–2:30 PM	Lunch				
	The Art and Science of Interdisciplinary Nutritional Therapy		Dr. Christopher Duggan		Dr Anura Kurpad Dr. Mainak Deb
3:45–4:00 PM	New learnings – Question and Answer session				
4:00–5:00 PM	The Role of Intravenous Lipid Emulsions in Intestinal Failure Associated Liver Disease		Dr. Kathleen Gura		Dr. Ashley D’Cruz Dr. Lekha Sreedharan
5:00–5:15 PM	Tea Break				
**Day 2–18th October 2025**					
Time	Session		Speaker(s)	Chairperson/moderator (s)	
8:50–9:00 AM	Welcome Remarks		Dr. Shalini Hegde		
9:00–9:15 AM	Maternal Morbidity and Impact on Birth Outcomes		Dr. Taruna Gupta	Dr. Harshpal Sachdev Dr Anura Kurpad	
9:15–9:30 AM	Paediatric Nutrition – First 1000 Days		Dr. Pratibha Dwarkanath	Dr. Harshpal Sachdev	
9:30–10:30 AM	Panel Discussion: Challenges in Preemies, Exclusive Breastfeeding		Dr. Suman Rao Dr. Ranjini Srinivasan Dr. Ranadip Chowdhury Dr. Sandhya Singh	Dr. Christopher Duggan Dr. Kiran M	
10:30–10:45 AM	Tea Break				
10:45–11:30 AM	Feeding Malnourished Children		Dr. Anura Kurpad	Dr. Christopher Duggan	
11:30–12:00 PM	Interactive Skills Session – Supplementary Nutrition Program		Dr. Tinku Thomas		
12:00–12:30 PM	Government Approach for Managing Malnutrition		Dr. Dakshayani B	Dr. Kanishka Das	
12:30–1:00 PM	Effect of Multi Domain Interventions during 1000+ Days		Dr. Ranadip Chowdhury	Dr. Harshpal Sachdev	
1:00–2:00 PM	Lunch				
2:00–2:15 PM	In-depth Knowledge Question and Answer session				
2:15–3:15 PM	Case Discussion				
2:15–3:15 PM	Biliary Atresia Stunting Cystic Fibrosis		Dr. Leekha Sreedharan Dr. Ranjini Srinivasan/Dr. Rebecca Raj Dr. Dhrithi Kodethoor/Dr. Malathi V		
3:15–3:45 PM	Insights Into the Role of a Clinical Pharmacist in the Nutrition Team		Mr. Mark Cowles	Sr. Jessie Margaret Saldanha	
3:45–4:00 PM	Tea Break				
4:00–4:30 PM	Interactive Session with All Faculty – PEN TO PAPER				
4:30–5:00 PM	Felicitation and Vote of Thanks				

The opening, and welcome remarks were delivered by Dr. Prasanna Kumar, Head of Department Paediatric Surgery, SJNAHS on Day 1 and Dr. Shalini Hegde, Associate Professor Paediatric Surgery, SJNAHS on Day 2. Dr. Ambily Sivadas, Assistant Professor, Division of Molecular Biology and Genetics, SJNAHS, Bengaluru, served as Master of Ceremony. During online sessions participant’s inquiries were collected through a chat box and were addressed by the chairpersons of the session.

## Summary of presentations

**Title: Setting the stage for Paediatric Nutrition.**

Speaker: Dr. Sarath Gopalan, Senior Consultant Paediatric Gastroenterologist, National President, Nutrition Society of India.

Chairpersons: Dr. Harshad Devarbhavi, Professor, Gastroenterology, St John’s Medical College; Dr. Gowrishankar, Professor, Pediatric Surgery, Indira Gandhi Institute of Child Health, Bengaluru.

Paediatric nutrition science emphasizes understanding and managing nutrition, both for the promotion of health as well as the prevention of disease. Useful concepts to guide nutritional interventions are the Estimated Average Requirement (EAR) (population-level planning), Recommended Dietary Allowance (RDA) (individual-level planning) and the Tolerable Upper Limit (population and individual level). There are various routes of nutrient delivery in paediatrics, such as oral, enteral and parenteral feeding. The talk also addresses popular food related myths in India and discusses innovative approaches in public health as well as clinical nutrition such as modulation of gut microbiota and chyme replacement therapy.

**Title: Paediatric Clinical Nutrition & Short Bowel Syndrome**.

Speaker: Dr. Dhanasekar Kesavelu, Senior Paediatric Gastroenterologist, Apollo Children’s Hospital, Chennai.

Chairpersons: Dr. Sanjay Rao, Professor, Pediatric Surgery, Narayana Health City, Bengaluru; Dr. Shilpa Varma, Clinical Nutritionist, Co-Secretary South Asian Association of Parenteral and Enteral Nutrition.

Short Bowel Syndrome (SBS) in children, is a challenging condition characterized by reduced absorptive capacity of the intestine. Enteral feeds when introduced early promotes intestinal adaptation. Complications include carbohydrate and fat malabsorption, bone demineralization and micronutrient deficiencies. Strategies to mitigate these include proactive management with non-absorbable antibiotics, early insertion of gastrostomy tubes, and vigilant infection control protocols. Advances in surgical management, such as the Serial Transverse Enteroplasty (STEP) and Longitudinal Intestinal Lengthening and Tailoring (LILT) procedures, may enhance bowel adaptation in selective cases. A proactive and individualized approach is crucial and significantly improves the long-term outcomes for children with SBS and intestinal failure.

**Title: Lines, Tubes, STEPS – The Surgeon’s Role in Supporting Nutrition**

Speaker: Dr. Biren Modi, Surgical Director, CAIR, Boston Children’s Hospital.

Chairpersons: Dr Ramesh Santhanakrishnan, Professor, Pediatric Surgery, Kshema Pediatrics; Dr. Padmalatha Kadamba, Professor and HOD, Pediatric Surgery, Ramaiah Memorial Hospital, Bengaluru.

Modern care emphasizes Interdisciplinary Rehabilitation Programs (IRPs) integrating surgeons, dieticians, and caregivers, improving patient survival outcomes from 72% to 87%. Maintaining central venous lines along with strict aseptic home care is vital since vascular access loss is life-limiting.

Over the decades, survival rates have improved from near-zero in the early 1900 s to over 95% today, with ongoing focus shifting toward quality of life. Multicentre studies now assess long-term psychosocial and family impacts, aiming for patient-centred outcomes. In conclusion, surgical excellence, nutrition science, and coordinated multidisciplinary care have transformed paediatric intestinal failure from a fatal condition into a manageable, survivable disease with meaningful quality of life.

**Panel discussion**

Moderator: Dr. Saleem Mammoo, Paediatric Surgeon, BEAR program, Sidra Hospital, Qatar.

Panel discussants for long-term Parenteral Nutrition (PN): Dr. Biren Modi, Dr. Anand Alladi, Dr. Amit Yelsangikar, Dr. Shubha AM.

Panel discussants for PN compounding: Dr. Gauri Krishna, Dr. Sanjay Rao, Dr. Antony Robert Charles, Dr. Manjiri S, Mr Mark Cowles (online), Ms. Fatima Azim (online).

There a few anecdotal instances of having children on long term PN in India. Barriers include absence of specialized teams such as interventional radiology to insert lines, limited protocols for long term management, non-availability of smaller sized central catheters for infants and a lack of repair kits. Currently long-term central lines are mainly placed in paediatric haemato-oncology services and rarely for nutritional indications. In contrast, adults are more likely to receive specialized and sustained PN due to better infrastructure and resource availability across centres.

PN compounding has distinct advantages including the ability to provide bespoke PN, and to reduce wastage. However, it has not gained traction in India because of high costs and absence of third-party reimbursements. Unlike countries such as the US and those in the Middle East where insurance or government support covers short bowel care, Indian families usually bear expenses themselves. Even for parents willing to be pay, specialized compounding facilities do not exist. Establishing such services would not only support short bowel syndrome but also benefit those with rare genetic and metabolic diseases who require customized nutrition.

**Title: The Art and Science of Interdisciplinary Nutritional Therapy (Friday Clinical Meeting)**.

Speaker: Dr. Christopher Duggan, Medical Director, CAIR, Boston Children’s Hospital.

Chairpersons: Dr. Anura Kurpad, Professor, Physiology, SJNAHS; Dr. Mainak Deb, Pediatric Surgeon, Rainbow Hospitals, Hyderabad.

The body’s ebb and flow response to trauma and critical illness varies considerably, making accurate measurements of energy requirements essential. Indirect calorimetry is an optimal method to measure Resting Energy Expenditure (REE) in the hospital, preventing both underfeeding and overfeeding when prescribing PN. Unlike REE, body composition is difficult to measure in the hospital setting but has much application in the research setting. Evidence from randomized control trials shows that unnecessary early PN in critically ill children increases infections and prolongs hospital stay. A dedicated transdisciplinary nutrition support team, to standardize care, reduces inappropriate PN use and improves nutrient delivery across ward and ICU settings. Examples from Boston Children’s Hospital demonstrate how structured teams, validated nutrition screening tools, and better bedside measurement practices improve the safety and effectiveness of nutrition care and support positive clinical outcomes.

**Title: The Role of IV Lipid Emulsions in Intestinal Failure–Associated Liver Disease (IFALD).**

Speaker: Dr. Kathleen Gura, Boston Children’s Hospital, President, American Society of Parenteral and Enteral Nutrition (Online).

Chairpersons: Dr. Ashley D’ Cruz, Professor, Pediatric Surgery, Narayana Health City, Bengaluru; Dr. Edwina Raj, Clinical Nutritionist, Aster CMI, Bengaluru.

Injectable lipid emulsions (ILE) are a source of essential fatty acids and an alternative to carbohydrates as a source of non-protein calories. Unfortunately, there is no ideal ILE. Despite traditional soybean-oil emulsions being rich in omega-6 fatty acids and phytosterols, they are associated with inflammation and cholestasis, particularly in neonates requiring prolonged parenteral nutrition. Comparison between available soybean, fish-oil, and mixed-oil formulations highlights differences in fatty-acid composition, vitamin E content, and hepatotoxic potential. Evidence shows that lipid restriction or mixed-oil emulsions protect against IFALD, while fish-oil–based emulsions reduces direct bilirubin and improves hepatic function. Fish oil lipid emulsion monotherapy at ≥ 1 g/kg/day can reverse IFALD, though high Paediatric End stage Liver Disease scores and delayed initiation reduces success. It is important to monitor individualized dosing an essential fatty acids status and using multidisciplinary approach as no ILE is universally optimal.

**Title: ICMR vision and current focus areas.**

Speaker: Dr. Taruna Gupta, Head of Developmental Research, ICMR.

Chairpersons: Dr. Harshpal Sachdev, Professor Paediatrics, Sitaram Bhartia Institute of Science and Research, New Delhi; Dr Anura Kurpad, Professor, Physiology, SJNAHS.

ICMR has a pivotal role in shaping biomedical and in public health researches across India. ICMR research infrastructure covers a variety of areas from vector-borne diseases to nutrition, and has established highly specialized research centres nationwide. There are collaborative efforts through global partnerships with leading institutions from the USA, Europe, and Asia, further strengthening India’s biomedical landscape.​

SAMPADA- a vision by ICMR, and one of its kind globally, collects robust data on diet, nutritional biomarkers, and physical activity, filling essential knowledge gaps regarding food insecurity and health across Indian states. ICMR’s strategic vision includes adapting guidelines for maternal nutrition through India-specific research, ensuring that policies are both evidence-based and contextually relevant. It is important to understand that a successful health innovation is a multidisciplinary approach with a nationwide implementation which constantly evaluates the public health needs.

**Title: Paediatric Nutrition (DBM/TBM) – The First 1000 Days.**

Speaker—Dr. Pratibha Dwarakanath Professor, Division of Nutrition, SJNAHS.

Chairpersons: Dr. Harshpal Sachdev, Professor Paediatrics, Sitaram Bhartia Institute of Science and Research, New Delhi; Dr. Anura Kurpad, Professor, Physiology, SJNAHS.

India’s paediatric nutrition landscape is marked by rising double and triple burden of malnutrition, underscoring the crucial role of the first 1000 days in shaping lifelong health. Malnutrition during this window heightens the of chronic diseases with global and Indian evidences, showing that Indian infants though smaller than western counterparts, carry similar body fat levels, predisposing them to metabolic obesity. Developmental Origins of Health and Disease framework reveals that rapid early-infancy weight gain, especially in small-for-gestational-age infants, increases risks of insulin resistance and cardiovascular disease. National data reflect persistent underweight along with rising childhood overweight. This strengthens the need for India-specific growth standards and initiatives such as the UNNATI Study. The current ICMR-CAR grant will use technological innovations such as stable isotope techniques and AI-based body fat assessment to inform holistic maternal-child nutrition strategies and to break the cycle of malnutrition and disease.

**Panel Discussion: Challenges in Preemies & LBW Infants; Exclusive Breastfeeding.**

Moderator: Dr. Kiran M; Professor, Paediatric Surgery, SJNAHS.

Chairperson: Dr. Christopher Duggan, Boston Children’s Hospital.

Speakers: Dr. Suman Rao, Dr. Ranjini Srinivasan, Dr. Ranadip Chowdhury, Dr. Sandhya Singh.

Despite institutional deliveries exceeding 90%, early initiation of breastfeeding remains low due to increased caesarean sections, limited rooming-in, inadequate antenatal counselling and maternal support. Working mothers especially in the unorganized sector, face major barriers in sustaining breastfeeding due to unsupportive workplaces. Misconceptions about milk sufficiency, cultural norms, limited family support add further complexity. Household constraints, shrinking family structures, poor hygiene and growing dependence on commercial foods impacts dietary diversity and adequacy. Healthcare challenges include insufficient training among frontline workers and suboptimal counselling practices. Addressing growth faltering, stunting, wasting, and underweight in infants require context-specific, multi-sectoral approaches that strengthen community and family support, promote responsive feeding, and ensure robust quality-assurance systems early childhood.

**Title: Feeding Malnourished Children.**

Speaker: Dr. Anura Kurpad, Professor, Physiology, SJNAHS.

Chairperson: Dr. Harshpal Sachdev, Professor Paediatrics, Sitaram Bhartia Institute of Science and Research, New Delhi.

Energy and protein needs for catch-up growth in children recovering from severe acute malnutrition (SAM) are provided at high rates given the tenfold mortality risk associated with this vulnerable group. Enthusiastic feeding, however, must be scientifically tailored to avoid complications like refeeding syndrome and fatty liver. Contrary to common assumptions, the case fatality rates are lower than global WHO estimates, and average weight gain is closer to 3 than 5 g/kg/day. Catch-up requirements depend on the type of tissue regained, and protein quality and bioavailability are important considerations. A refined approach is required to feed in malnutrition, balancing the need for catch-up growth against metabolic safety and long-term health.

**Title: Interactive Skills Session – Supplementary Nutrition Program (ICDS).**

Speaker: Dr. Tinku Thomas, Professor and Head of Biostatistics, SJNAHS.

There is need to strengthened India’s Supplementary Nutrition Program under Integrated Child Development Services (ICDS) scheme by moving from calorie-centric approach to nutrient-dense diets for pre-school children and pregnant/lactating women. POSHAN 2.0 prioritizes maternal nutrition, Infant Young Child Feeding, and targeted strategies for moderate and severe acute malnutrition, aiming to reach nearly 100 million beneficiaries.

Updated national guidelines specify flexible energy, protein, fat, and micronutrient norms 400–800 kcal and 15–30 g protein for young children, and up to 600 kcal and 22–25 g protein for pregnant and lactating women, with higher needs for SAM-affected children. A major innovation is a web-based linear programming tool that helps states design cost-effective Take-Home-Ration and Home-Cooked-Meal menus using diverse local foods. This data-driven approach, adopted across several states empowers public-nutrition interventions improves outcomes for vulnerable populations. A hands on session of this tool was conducted.

**Title: Government Approaches to Managing Malnutrition – CMAM Program (Community Management of Acute Malnutrition).**

Speaker: Dr. Dakshayani B, Associate Professor, Paediatrics, Bangalore Medical College.

Chairperson: Dr. Kanishka Das, Professor, Paediatric Surgery, AIIMS, Bhubaneshwar.

The government’s strategy to combat malnutrition relies on a multipronged, cross-sectoral approach that recognizes malnutrition as a complex issue shaped by social, health, and environmental factors. Key initiatives ICDS, Mid-Day Meal, and Poshan Abhiyaan target nutritional needs across the life course, with special focus on children, pregnant and lactating women, and adolescent girls. These programs aim to improve access to quality supplementary nutrition, strengthen breastfeeding and infant and young child feeding practices, and enhance frontline worker capacity through training and accountability.

Persistent challenges include gaps in service delivery, weak monitoring, limited departmental convergence, and deep-rooted social determinants such as poverty, illiteracy, and gender inequality. Stronger community participation, effective behaviour-change communication, and technology-enabled monitoring are critical for improving impact. Ultimately, sustained political commitment, coordinated action, and adequate resources remain essential to achieving lasting progress in reducing malnutrition and improving population health.

**Title: Effect of Multi-Domain Interventions During the First 1000 + Days on Neurodevelopment**.

Speaker: Dr. Ranadip Chowdhury, Deputy Director and Scientist, Society of Applied Sciences, New Delhi.

Chairperson: Dr. Anura Kurpad, Professor, Physiology, SJNAHS.

The “Multi-Domain Interventions during 1000 Plus Days” trial highlights the strong impact of integrating health, nutrition, psychosocial care, and WaSH interventions from preconception through early childhood. Because low birth weight and stunting have multifactorial causes, meaningful improvement requires addressing multiple determinants across life stages. The WINGS trial showed that combining preconception and pregnancy/childhood interventions produced the largest gains, including up to a 31% reduction in small-for-gestational-age births and over 50% reduction in stunting at two years. Children also demonstrated better cognitive, language, motor, and socio-emotional development, with a 35%–70% lower risk of moderate to severe neurodevelopmental delay. These findings emphasize early, continuous, and multisectoral action, particularly in high-risk communities, and call for scalable implementation within public health systems.

## Case discussions

### Case 1: Biliary atresia

Speaker: Dr. Lekha Sreedharan, Clinical Nutritionist, Apollo Hospitals, Chennai, Vice-President, IAPEN.

This rare congenital obstruction of the bile ducts rapidly progresses to liver failure without early intervention. Persistent jaundice beyond 14 days is a key warning sign, prompting urgent evaluation, as biliary atresia accounts for about half of paediatric liver transplants. The Kasai procedure is the initial treatment, but outcomes vary some infants clear jaundice while others progress to cirrhosis and ultimately require transplant. Nutritional care is central, as impaired absorption, high metabolic demands, and chronic liver disease drive malnutrition. Regular monitoring of weight, length, and mid-arm circumference guides high-calorie (~130% of energy intake), high-protein (2–2.5 g/kg/day) nutrition using medium chain triglycerides (MCT) -rich formulas, supplements, and fortified feeds. Post-transplant, frequent meals, continued supplementation, and tube or parenteral nutrition when needed support growth, prevent deficiencies, and improve overall outcomes.

### Case 2: Stunting

Speakers: Dr. Rebecca Raj, Professor and HOD, Division of Nutrition, SJNAHS and Dr. Ranjini S, Professor, Paediatrics, SJNAHS.

Chronic undernutrition and stunting remain major public health challenges in India, affecting children’s growth, cognitive development, and long-term health. Stunting height-for-age below two SD of WHO standards reflects prolonged nutritional deprivation and socioeconomic inequities. Though rates have declined, the latest National Family Health Survey (NFHS −5) still reports 35.5% of children stunted, with a higher burden in rural than urban areas. A Karnataka case example highlights common risks: low birth weight, poor maternal nutrition, limited antenatal care, recurrent infections, delayed complementary feeding, and food insecurity. These lead to delayed development, higher infection risk, reduced productivity, and intergenerational consequences. Management requires coordinated action: national programs that strengthen food and nutrition security, community support through anganwadis, improved hygiene and sanitation, and family-level practices including diverse, nutrient-rich diets, regular health checks, and immunization.

### Case 3: Cystic fibrosis

Speakers: Dr. Dhrithti Kodethoor, Assistant professor, Paediatrics, SJNAHS and Dr Malathi V, Clinical Nutritionist, Rainbow Hospitals, Bangalore.

Cystic fibrosis (CF), an autosomal recessive disorder with major lung and gastrointestinal involvement, remains under-recognized in India. Malnutrition affects up to half of children with CF and is linked to poorer lung function, frequent infections, and higher mortality. Nutritional failure results from pancreatic insufficiency, fat malabsorption, chronic inflammation, increased energy needs, and micronutrient losses. Current ESPEN–ESPGHAN–ECFS guidelines recommend a high-calorie, high-fat, protein-rich diet; optimized pancreatic enzyme replacement; and supplementation of fat-soluble vitamins, sodium, calcium, and trace elements. Regular growth monitoring and personalized feeding plans are essential. Case examples illustrate challenges such as feeding intolerance, gastro-esophageal reflux disease, and psychosocial barriers, with some children requiring enteral feeding. Early detection of CF-related diabetes with annual oral glucose tolerance test and carbohydrate counseling is vital. Proactive nutrition care improves growth, delays respiratory decline, and enhances quality of life.

**Title: Insights into the Role of a Clinical Pharmacist in the Nutrition Team.**

Speaker: Mr. Mark Cowles, Clinical Pharmacist, Sidra Hospital, Qatar (online).

Chairperson: Sr. Jessie Saldanha, HOD pharmacy, SJNAHS.

PN for high-risk paediatric patients is complex and must always be individualized. The focus should be on the child, not just the lab numbers. Dextrose, lipids, and electrolytes each need thoughtful adjustments based on clinical goals, and blood sampling is timed correctly to avoid misinterpretation during PN cycles. The energy–nitrogen ratio is tailored for paediatric patients needing lipids, who often require higher ratios than stable adults. If something is not technically feasible, it will not work clinically, so operational practicality matters. Monitoring always includes trace elements, vitamins, and frequent reassessment of dosing weight using accurate growth charts. Managing PN at scale demands strong coordination, and technologies like barcoded labels and advanced mixing pumps to enhance safety. PN therapy is dynamic, and a child needs to be continually reassessed to optimize outcomes.

## Conclusion

This symposium successfully united clinical, community paediatrics and health policy to emphasize the central role of nutrition in improving survival, growth, and neurodevelopment of children across India. Advances shared in the clinical management of complex surgical conditions demonstrated how expert, multidisciplinary nutrition care can transform outcomes for the most vulnerable children. Equally, the sessions emphasized that improving nutrition for all children requires strong community-based services, family support, and empowered frontline workers. By aligning clinical excellence with robust public health systems and progressive nutrition policies, the symposium reinforced that meaningful and sustained improvements in paediatric nutrition depend on coordinated action from the hospital ward to the household — supported by evidence, innovation, and multisectoral commitment.

## Abbreviations

CAR: Centre of Advanced Research
CF: Cystic Fibrosis
EAR: Estimated Average Requirement
ECFS: European Cystic Fibrosis Society
ESPEN: European Society for Clinical Nutrition and Metabolism
ESPGHAN: European Society for Paediatric Gastroenterology, Hepatology and Nutrition
ICDS: Integrated Child Development Services
ICMR: Indian Council of Medical Research
IDA: Indian Dietetic Association
IFALD: Intestinal Failure–Associated Liver Disease
ILE: Intravenous Lipid Emulsion
IAPEN: Indian Association for Parenteral and Enteral Nutrition
IRP: Intestinal Rehabilitation Program
LBW: Low Birth Weight
LILT: Longitudinal Intestinal Lengthening and Tailoring
MCT: Medium-Chain Triglyceride
NFHS: National Family Health Survey
PN: Parenteral Nutrition
REE: Resting Energy Expenditure
RDA: Recommended Dietary Allowance
SAM: Severe Acute Malnutrition
SAS: Society of Applied Sciences
SBS: Short Bowel Syndrome
SJNAHS: St John’s National Academy of Health Sciences
STEP: Serial Transverse Enteroplasty
TBM: Triple Burden of Malnutrition
WaSH: Water, Sanitation and Hygiene
WHO: World Health Organization
